# How Long Do Microorganisms Survive and Persist in Food? A Systematic Review

**DOI:** 10.3390/microorganisms13040901

**Published:** 2025-04-14

**Authors:** Eric S. Donkor, Famous K. Sosah, Alex Odoom, Bernard T. Odai, Angela Parry-Hanson Kunadu

**Affiliations:** 1 Department of Medical Microbiology, University of Ghana Medical School, Korle Bu, Accra P.O. Box KB 4236, Ghana; famoussosah1@gmail.com (F.K.S.); alexodoom2018@gmail.com (A.O.); 2 Radiation Technology Centre, Biotechnology and Nuclear Agriculture Research Institute, Ghana Atomic Energy Commission, Accra P.O. Box LG 80, Ghana; bernywept@gmail.com; 3 Department of Nutrition and Food Science, University of Ghana, Accra P.O. Box LG 134, Ghana; aparry-hanson@ug.edu.gh

**Keywords:** persistence, survival, pathogens, environmental factors, food supply chain, food safety, review

## Abstract

Foodborne illnesses caused by microorganisms pose a significant threat to public health. Understanding the survival and persistence of these microorganisms in various food matrices is crucial for developing effective control strategies. This systematic review aims to address the current knowledge gaps related to the duration of survival and persistence of microbial pathogens in food, as well as the impact of external environmental conditions on their viability. A comprehensive search was conducted across major databases, including studies published until 3 June 2024. The PRISMA guidelines were followed to ensure a systematic and transparent approach. Foodborne bacteria, such as *Salmonella* spp., *Listeria monocytogenes*, and *Escherichia coli* O157:H7, were found to persist for extended durations, ranging from days to over a year. The mean duration of persistence for all of the bacteria was 246 days, whereas the survival duration was 16 days. Bacterial survival and persistence were significantly influenced by temperature, with warmer conditions (>25 °C) generally supporting longer persistence. Relative humidity also played a role, with low-humidity environments (<50% RH) favouring the survival of pathogens like *Listeria monocytogenes* and *Escherichia coli*. In contrast, viruses, such as hepatitis A virus and Human norovirus, showed only survival patterns, with average durations of 21 days and temperature being the primary environmental factor influencing their survival. Overall, this review provides evidence that a wide range of microbial pathogens, including *Escherichia coli* O157:H7, *Salmonella* spp., *Listeria monocytogenes*, and the hepatitis A virus, can survive and persist in food for prolonged periods, leading to potential harm. These insights underscore the necessity of stringent food safety measures and continuous monitoring to mitigate the risks posed by these resilient pathogens, contributing to a safer and more secure food supply chain.

## 1. Introduction

The high and increasing incidence of foodborne illnesses, coupled with the appearance of new foodborne pathogens and the return of previously controlled ones, has made food safety a very important public health issue. According to estimates from the World Health Organization, there are approximately 600 million food-related illnesses annually, resulting in 420,000 deaths and the loss of 33 million healthy life years (disability-adjusted life years (DALYs)) [1]. Foodborne illnesses are also associated with a high economic burden. For example, the USDA Economic Research Service (ERS) estimated that the cost of foodborne illness in the United States was $17.6 billion in 2018; this was an increase of $2 billion from 2013, when the cost was estimated to be $15.5 billion [2]. In Australia, the cost of foodborne illnesses is estimated at $1.289 billion per year [3], while in New Zealand, the cost is $86 million [4]. This substantial global health and economic toll underscores the critical need for improved food safety practices and interventions to effectively mitigate the risks posed by persistent foodborne pathogens.

The common causes of foodborne illnesses include bacteria such as *Listeria monocytogenes*, *Escherichia coli*, and *Salmonella*; fungi such as *Penicillium* and *Aspergillus*, which produce harmful mycotoxins; viruses such as norovirus, hepatitis A and E, and rotavirus; and parasites such as *Trichinella spiralis*, *Toxoplasma gondii*, and *Cyclospora* [5,6]. These pathogens can be introduced into food at various stages, including during its production, processing, storage, and handling [7]. Several lines of evidence show that some pathogens can survive by entering a dormant or injured state, allowing them to recover or persist [8,9,10]. Pathogen persistence in food depends on many factors, such as their physical and microbial habitats, transmission routes, stress adaptations, and genetic determinants, and can cause repeated food contamination [11,12,13]. Pathogens can modify their cellular structures, metabolic processes, and virulence factors to thrive in hostile food environments, such as at extreme temperatures, in high-salt or low-moisture conditions, and at acidic pH levels [14,15,16]. This remarkable adaptability allows them to persist for extended durations, posing significant challenges for food safety and public health.

Gaining insight into the survival durations and persistence of different pathogens in various types of food and the influence of environmental factors is essential for developing targeted control strategies. Despite the plethora of primary research data on the subject and its significance, a systematic review that provides comprehensive information to guide preventive, control, and management efforts has yet to be undertaken. This systematic review aims to address this knowledge gap by determining the duration of pathogen survival and persistence in food, as well as the role of environmental factors in modulating their viability, to support the development of more robust food safety strategies.

## 2. Materials and Methods

### 2.1. Protocol and PRISMA Guidelines

The review protocol was registered in the Open Science Framework (OSF) repository and can be accessed via this link: https://doi.org/10.17605/OSF.IO/75GY4 (accessed on 13 January 2025). To ensure a systematic and transparent approach to the literature search and review, the Preferred Reporting Items for Systematic Reviews and Meta-Analyses (PRISMA) guidelines [17] were followed. The PRISMA guidelines offer a detailed checklist and flow diagram that assist in record identification, screening, and evaluation.

### 2.2. Search Strategy

A comprehensive search was conducted across major databases, including PubMed, Web of Science, and Scopus, using the following search terms: (“persistence” OR “survival”) AND (“pathogen” OR “bacteria” OR “fungi” OR “viruses” OR “parasites”) AND (“food” OR “fruits” OR “vegetables” OR “fresh produce” OR “Seafood” OR “eggs” OR “dairy” OR “meat” OR “milk”). Additionally, the reference lists of the identified articles were examined to identify any additional relevant publications. The search spanned studies published until 3 June 2024, with no restrictions on the study period and geographical location. Only studies published in English were included to maintain consistency and feasibility in the data extraction and analysis, as most of the studies were in English. The decision to not apply geographical restrictions was a deliberate choice made to capture a more comprehensive and representative understanding of the survival and persistence of foodborne pathogens globally. The primary focus was studies that provided experimental evidence concerning the duration of pathogen survival and persistence in any type of food. These studies used controlled laboratory experiments, field trials, or other empirical investigations to directly observe and measure the survival and/or persistence of foodborne pathogens in various food matrices or environmental conditions.

### 2.3. The Study Selection Process

A two-stage screening process was employed to identify relevant studies for inclusion in the review. Initially, the titles and abstracts of the identified studies were reviewed to eliminate duplicates and irrelevant articles. Next, the full texts of the remaining studies were thoroughly examined against predefined inclusion and exclusion criteria to determine their eligibility. Studies providing original data on pathogen survival or persistence on food were included in the review, and the results were extracted. Studies focusing on pathogen survival on food contact surfaces were excluded because they were outside the scope of this review. Similarly, studies that detected only the presence of pathogens without estimating the duration of their survival or persistence were excluded, although they were screened for relevant information. For the purposes of this review, survival refers to the ability of a pathogen to remain alive and viable over a specific period, typically ranging from a few days to less than three months, whereas persistence is defined as the repeated isolation of a pathogen over a 3-month period, maintaining the same molecular or genetic characteristics. Similarly, biofilm formation is acknowledged as a crucial factor in providing a stable environment for pathogen survival and persistence.

Two reviewers, A.O. and F.K.S., independently conducted the screening process, adhering to the predefined criteria, with a third reviewer consulted to resolve any disagreements. The third reviewer ensured consistency with the predefined criteria, provided an unbiased perspective to resolve conflicts, and leveraged their expertise to make informed decisions in complex cases.

Mendeley Desktop version 1.19.8 was utilized to manage the search results and identify duplicate records.

### 2.4. Data Extraction

The data were independently extracted by the two reviewers via a Microsoft Excel 2019 spreadsheet (Version 2412). The information extracted from the eligible articles included the authors, the pathogens studied, the food type or sample, the duration of pathogen survival or persistence, and the external conditions tested.

### 2.5. Quality Assessment

The risk of bias in each study was evaluated via the Cochrane ROB2 tool [18], and the results were visually presented via the robvis tool [19]. The Cochrane ROB2 tool is a widely recognized framework for assessing potential sources of bias in randomized controlled trials, commonly used in food safety research. The robvis tool further enhanced the analysis by visualizing the risk of bias assessments, making it easier to identify areas of concern and interpret the review’s findings in relation to the overall methodological quality of the studies. This assessment tool examines five key areas of bias, namely randomization, deviations from the planned interventions, missing outcome data, outcome measurement, and the selection of reported results. The two reviewers independently assigned a classification of low bias, high bias, or some risk of bias to each domain, and any discrepancies were resolved through consultation with the third reviewer. A study was considered to have a low risk of bias if all domains received a low-risk classification, to have a high risk if at least one domain was rated as high-risk, and for some concerns to be present if there were issues identified in one or more domains.

### 2.6. Data Synthesis

Descriptive statistics were used to summarize the key findings on the pathogen survival and persistence durations. The mean durations, as well as the standard deviation values, were calculated. The findings were synthesized thematically and conveyed through textual narratives, tables, and figures.

## 3. Results

### 3.1. Search Results

**Figure 1:**
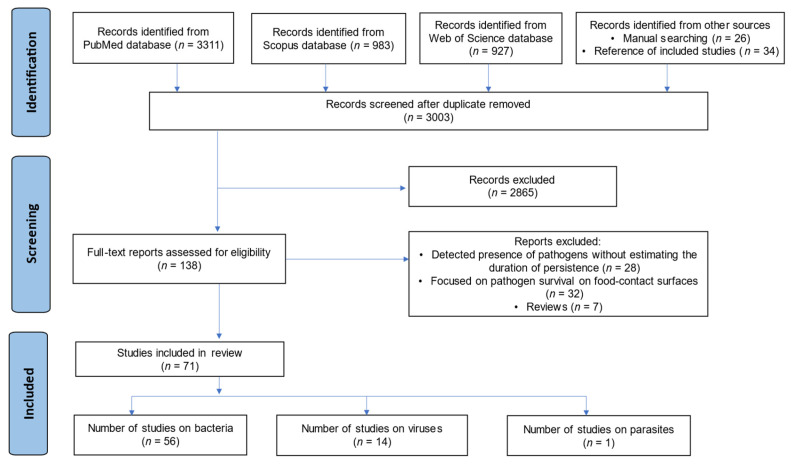
PRISMA flow diagram for the identification, screening, and evaluation of the articles included in the study.

Initially, a total of 5281 records were identified from various databases, including PubMed (*n* = 3311), Scopus (*n* = 983), and Web of Science (*n* = 927), and from a citation search of relevant articles (*n* = 60). After removing duplicates (*n* = 2278), the titles and abstracts of the remaining 3003 records were screened. Among these, 2865 articles were excluded because they did not meet the established inclusion criteria for the review. Subsequently, 138 full-text articles were evaluated for eligibility, resulting in 71 articles [20,21,22,23,24,25,26,27,28,29,30,31,32,33,34,35,36,37,38,39,40,41,42,43,44,45,46,47,48,49,50,51,52,53,54,55,56,57,58,59,60,61,62,63,64,65,66,67,68,69,70,71,72,73,74,75,76,77,78,79,80,81,82,83,84,85,86,87,88,89,90] that met the inclusion criteria for the review, with 56 focusing on bacteria [20,21,22,23,24,25,26,27,28,29,30,31,32,33,34,35,36,37,38,39,40,41,42,43,44,45,46,47,48,49,50,51,52,53,54,55,56,57,58,59,60,61,62,63,64,65,66,67,68,69,70,71,72,73,74,75], 14 on viruses [76,77,78,79,80,81,82,83,84,85,86,87,88,89], and 1 on parasites [90] (Figure 1).

### 3.2. Survival and Persistence of Bacteria

The survival and persistence of bacteria in various food samples were reported in 56 (78.9%) of the studies included in the review. These studies identified 13 different genera of bacteria that survived and persisted on food for varying durations (Table A1).

#### 3.2.1. Frequency of Bacteria

**Figure 2:**
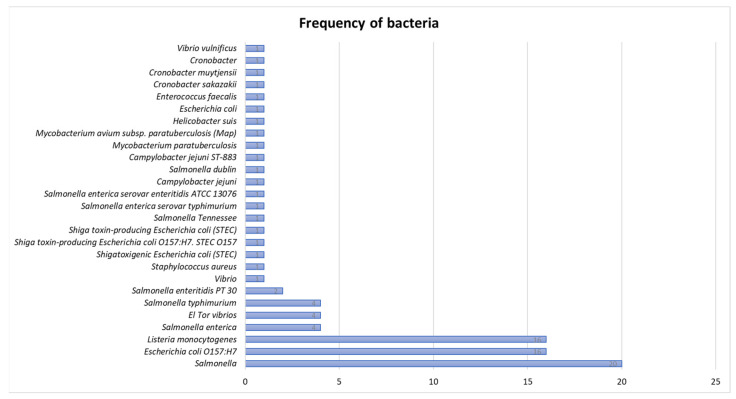
Frequency of bacterial pathogens in different time ranges in various food samples.

*Salmonella* was the most frequently identified bacteria, appearing 20 times in different time ranges in various food samples, followed by *Listeria monocytogenes* and *Escherichia coli* O157:H7, each with a frequency of 16. Less frequently identified bacteria included *Staphylococcus aureus*, *Vibrio*, *Enterococcus faecalis*, *Helicobacter suis*, and *Mycobacterium avium subsp. paratuberculosis* (Map) (Figure 2).

#### 3.2.2. Survival and Persistence Durations of Bacteria

Bacteria can persist for long durations, often several months to a year. The mean duration of persistence for all of the bacteria was 246 days, whereas the survival duration was 16 days (Table 1). *Salmonella* spp. had the longest persistence duration, lasting 36 months (approximately 1095 days) [57].

#### 3.2.3. Common Conditions Under Which Bacteria Show Survival and Persistence

##### Influence of Temperature on Bacterial Survival and Persistence

Bacteria are capable of surviving and persisting across a wide range of temperatures, with warm conditions supporting the longest persistence durations (Table 2). Persistence is frequently observed at moderate temperatures (23–25 °C), although some cases demonstrate persistence even at high temperatures (40 °C). For example, certain bacteria, such as *Salmonella*, have persisted for up to 36 months [57], showcasing their adaptability to high temperatures. Persistence is also observed at lower temperatures, including 4 °C and even as low as −24 °C. Bacteria such as *Campylobacter jejuni*, *Escherichia coli* O157:H7, and *Listeria monocytogenes* have been found to survive for extended periods (21–30 days), indicating that low temperatures slow bacterial growth but do not eliminate their survival. At moderate temperatures (10–25 °C), most bacteria, including *Escherichia coli* and *Salmonella*, can survive for days to weeks, suggesting that these conditions are conducive to bacterial survival. However, at higher temperatures (>25 °C), the duration of bacterial survival generally decreases.

##### Influence of Humidity on Bacterial Survival and Persistence

Bacteria exhibit varying survival and persistence durations under different humidity conditions. In high-humidity environments (>70% RH), *Salmonella* and *Listeria monocytogenes* persisted for up to 365 days [60] and 180 days [47], respectively, indicating that high humidity promotes their persistence. Interestingly, in low-humidity environments (<50% RH), *Listeria monocytogenes* exhibited a longer persistence period of 336 days [49]. Similarly, *Escherichia coli* O157:H7 persisted for up to 12 months in low-humidity conditions (<50% RH) [28]. In contrast, *Enterococcus faecalis* survived for only 7 days in medium-humidity environments (50–70% RH) [25].

##### Influence of pH on Bacterial Survival and Persistence

*Escherichia coli* O157:H7 can survive for 30 days at a low pH (2.51–3.26) [34], but its survival duration reduces to 21 days at a pH of 5.5 [38], showing that some bacteria can endure well in higher acidic environments.

##### Influence of Food Matrix on Bacterial Survival and Persistence

*Listeria monocytogenes* persists longer, for over a year, in low-moisture foods such as walnut kernels, raw peanuts, and pecan kernels [36,37] than in high-moisture foods such as raw juice [26,29]. *Salmonella* and *Escherichia coli* O157:H7 demonstrated similar trends, persisting for over a year in high-moisture foods [36,37]. Generally, dairy products and processed foods tend to support both bacterial survival and persistence. Fruits and vegetables primarily show survival whereas nuts and seeds often exhibit persistence, likely due to their low moisture content.

### 3.3. Survival and Persistence of Viruses

Fourteen studies examined the survival and persistence of viruses in various food samples, with sixteen distinct virus species found to survive in different types of food (Table A2).

#### 3.3.1. The Frequency of Viruses

**Figure 3:**
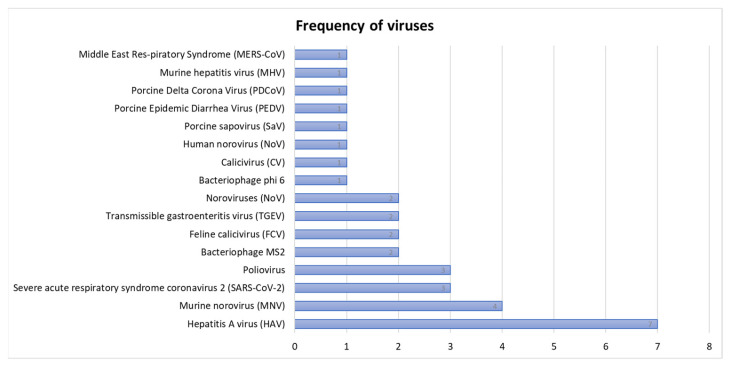
Frequency of viral pathogens at different time ranges in various food samples.

Hepatitis A virus (HAV) was the most frequent virus, appearing seven times at different durations in various food samples (Figure 3).

#### 3.3.2. Survival Durations of Viruses

Contrary to bacteria which exhibited both survival and persistence, viruses showed only survival. The average duration of virus survival across different samples is approximately 21.29 days, with a standard deviation of 14.97 days. This duration ranges from a minimum of 2 days to a maximum of 56 days, indicating significant variability among different types of samples.

#### 3.3.3. Common Conditions Under Which Viruses Show Survival

##### Influence of Temperature on Virus Survival

Temperature is the most significant factor affecting virus survival, with cooler conditions generally extending its duration. The most frequent conditions for virus survival are low/refrigerated temperatures and room temperature (Table 3). At low temperatures, viruses can survive for extended periods, ranging from a few days to several weeks. For example, hepatitis A virus can last up to 4 weeks. Similarly, room temperature allows for significant survival durations, often up to 6 weeks. Frozen temperatures support survival for 15 days. However, higher temperatures, particularly those above what are considered “warm conditions”, tend to reduce survival times, with viruses surviving for only 2 weeks at such elevated temperatures.

##### Influence of Humidity on Virus Survival 

Whereas temperature is the most significant factor affecting virus survival, humidity levels do not show a significant impact on survival duration. For instance, bacteriophage MS2 survived on oysters and fresh peppers for 2 weeks at various temperatures (4, 15, 25, and 40 °C) with relative humidity levels of 50% and 70%. Hepatitis A virus (HAV) exhibited a similar survival pattern under the same conditions as bacteriophage MS2 [76]. A similar trend was observed in Middle East respiratory syndrome (MERS-CoV), which survived on apples and tomatoes for 72 h at 22 °C with a relative humidity of 30–40%. Severe acute respiratory syndrome coronavirus 2 (SARS-CoV-2) also survived for 72 h under similar conditions to MERS-CoV [83], indicating that relative humidity does not drastically affect the survival duration of viruses.

##### Influence of pH on Virus Survival

pH levels do not show a significant impact on the survival duration of viruses. For instance, viruses like hepatitis A virus survived for 4 weeks at room temperature on marinated mussels [79]. However, even in the presence of a pH of 3.75, the survival of hepatitis A virus for 4 weeks suggests that acidic conditions do not significantly affect survival time [80].

##### Influence of Food Matrix on Virus Survival

There was a noticeable correlation between the type of sample and the duration of virus survival. Viruses tend to survive longer on certain types of samples, such as feed ingredient matrices and seafoods and cereal, compared to fresh produce. The longest survival was observed on alfalfa seeds [81].

### 3.4. The Survival and Persistence of Protozoa Parasites

Unlike bacteria and viruses, only one of the included studies investigated the survival and persistence of parasites in food. *Cryptosporidium parvum* was found to survive on lamb’s lettuce for a duration of two months [90].

### 3.5. Risk of Bias

**Figure 4:**
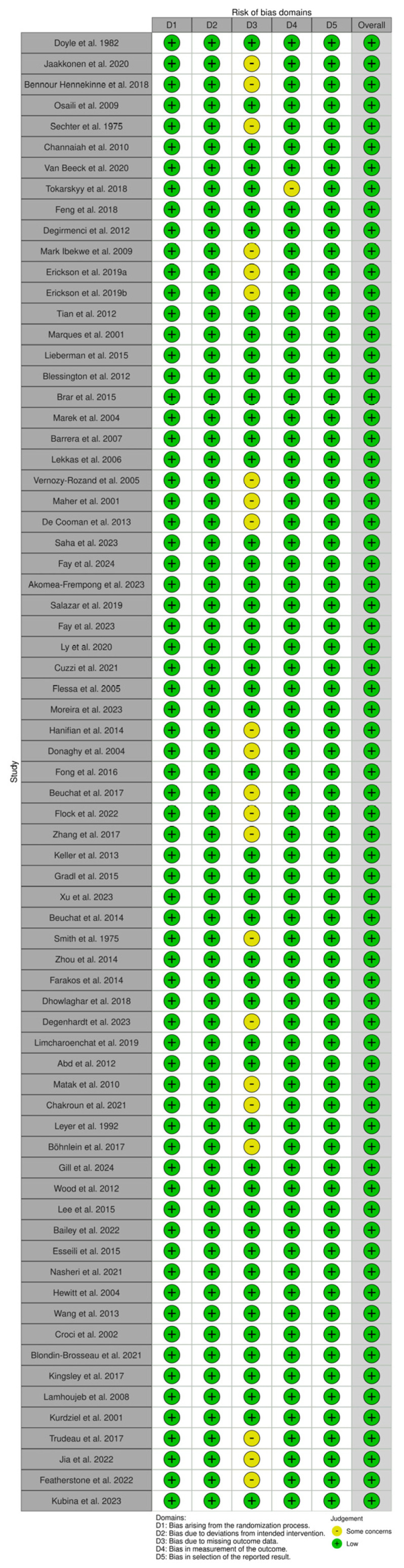
Assessment of bias of the included studies using the Cochrane Risk of Bias [20,21,22,23,24,25,26,27,28,29,30,31,32,33,34,35,36,37,38,39,40,41,42,43,44,45,46,47,48,49,50,51,52,53,54,55,56,57,58,59,60,61,62,63,64,65,66,67,68,69,70,71,72,73,74,75,76,77,78,79,80,81,82,83,84,85,86,87,88,89,90].

The risk of bias for the 71 studies included in this systematic review was assessed using the robvis tool, as shown in Figure 4. This tool categorizes bias into three levels: low-risk (green), some concern (yellow), and high-risk (red). The majority of studies were rated as having a low risk of bias. However, a few studies showed some concerns, particularly in the domains of performance and detection bias. These concerns were primarily due to a lack of blinding and incomplete outcome data. The overall low risk of bias across all domains suggested that the studies were methodologically sound and reliable.

## 4. Discussion

The survival and persistence of pathogens in food pose a serious threat to food safety and public health. We aimed to provide valuable information regarding the duration for which pathogens can remain viable on different food items. One notable knowledge gap identified in this review was the limited number of studies focused on the survival and persistence of protozoan parasites and fungi in food matrices. There is a substantial body of evidence (78.9%) on the behavior of bacterial pathogens, such as *Salmonella*, *Listeria monocytogenes*, and *Escherichia coli*; however, the data available for protozoan parasites, like *Cryptosporidium* and *Giardia lamblia*, as well as foodborne fungi, such as *Aspergillus* spp., *Penicillium* spp., and *Fusarium* spp., are relatively scarce. This lack of research on non-bacterial foodborne pathogens is concerning, as these microorganisms can pose significant public health risks and have the potential to survive and persist in food production and processing environments. Protozoan parasites, for example, are known to form environmentally resistant cysts that can withstand a range of adverse conditions, potentially allowing them to remain viable in contaminated food and water sources for extended periods. For instance, *Cryptosporidium parvum* is globally associated with foodborne illnesses, which account for more than 8 million cases annually [91]. Similarly, many fungal species are adept at adapting to diverse ecological niches and may develop specialized survival strategies, such as the production of mycotoxins, which can withstand heat and processing, thereby posing a risk in both raw and processed foods [92].

Compared to bacteria, only 19.7% of the included studies detected the survival and persistence of viral pathogens. This may be due to difficulties in the detection and quantification of the viral genome [93]. The duration of bacterial species in food samples was greater than that of viral species. Generally, bacteria persist for longer durations on food, up to 36 months, than viruses, which persist for up to 60 days. However, the shortest survival duration was greater for viruses at 72 h than for bacteria at 24 h. *Salmonella* is a major concern among pathogens because of its ability to persist for extended periods, as observed in this review, making it one of the most prevalent zoonotic foodborne pathogens and a significant threat to global public health [94]. Salmonella contamination in food products poses a significant risk to consumers. For instance, it causes both typhoid fever and gastroenteritis, with nontyphoidal *Salmonella* (NTS) serovars being associated with the latter [95]. According to the World Health Organization’s estimation in 2010, there were approximately 153 million NTS infections worldwide, leading to 56,969 fatalities, with almost half of these cases resulting from foodborne transmission [95]. Additionally, in 2018, *Salmonella* was responsible for more than half of the reported foodborne illness outbreaks in the European Union [96]. *Salmonella* is transmitted to humans throughout the entire food production process, from farm to fork, primarily through the consumption of contaminated animal- and plant-based foods [97].

One of the key factors influencing pathogen persistence is the surrounding environment. Most of these studies examined the influence of temperature on the persistence of foodborne pathogens. Temperature can affect the growth, reproduction, and overall survival of these microorganisms [98]. Pathogens are typically sensitive to high temperatures; however, higher temperatures within their optimal growth range facilitate faster replication and increase the contamination risk [99]. Both bacteria and viruses were found to survive at relatively high temperatures, with bacteria surviving up to 40 °C. Specifically, *Salmonella*, hepatitis A virus, and murine norovirus presented increased heat resistance. On the other hand, viruses such as poliovirus were able to survive at temperatures as low as −20 °C, whereas bacteria such as *Escherichia coli*, *Listeria monocytogenes*, and *Salmonella* survived at −24 °C. Refrigeration at approximately 4 °C is generally effective in slowing the growth of most pathogens and prolonging the shelf-life of food products. However, *Escherichia coli* O157:H7 is an exception because of its ability to survive and withstand refrigeration conditions compared with storage conditions at room temperature [100]. This particular strain of *Escherichia coli* is strongly associated with foodborne disease outbreaks, especially those that are linked to the consumption of contaminated leafy green vegetables [101]. Moreover, factors such as humidity and air quality affect the growth and persistence of pathogens [102,103]. Few studies examined the influence of relative humidity on pathogen survival and persistence in/on food. Typically, elevated humidity provides an ideal setting for the growth and survival of pathogens, especially in foods with a high moisture content [104]. At low relative humidity levels, many foodborne pathogens must contend with the challenge of water loss and desiccation. In response, these microorganisms have developed specialized mechanisms to maintain cellular homeostasis and prevent dehydration-induced damage. For example, *Salmonella enterica* has been shown to upregulate the production of trehalose, a disaccharide that can act as a compatible solute, to protect cellular structures and proteins from the detrimental effects of water loss [15].

Apart from environmental influences, the underlying molecular and physiological mechanisms of foodborne pathogens can also significantly impact their survival and persistence in various food matrices. For instance, the ability of *Listeria monocytogenes* to survive and even thrive at refrigeration temperatures is often attributed to its capacity to adapt and express specialized survival strategies. One such mechanism is the formation of resilient biofilms, which can provide enhanced protection against environmental stressors, such as low temperatures, desiccation, and antimicrobial agents. *Listeria monocytogenes* has been shown to produce exopolysaccharides such as poly-β-(1,4)-N-acetylmannosamine and teichoic acids, as well as surface proteins including InlA, BapL, and PlcA that facilitate the attachment and development of biofilms on food contact surfaces, allowing the pathogen to persist in the processing environment [14]. Similarly, the production of heat shock proteins is another key physiological response that enables pathogens like *Salmonella enterica* to withstand thermal challenges. These proteins, such as DnaK, GroEL, and ClpB, help stabilize cellular structures, facilitate the refolding of denatured proteins, and maintain essential metabolic functions, thereby enhancing pathogens’ resilience in the face of heat stress [16]. This adaptive mechanism allows *Salmonella* to survive pasteurization temperatures and continue to pose a risk in heat-treated food products. Moreover, changes in the composition and fluidity of the cell membrane can also contribute to temperature tolerance in foodborne pathogens. For example, *Campylobacter jejuni* has been observed to modify the ratio of saturated to unsaturated fatty acids in its cell membrane in response to low temperatures, maintaining membrane integrity and fluidity to ensure continued metabolic activity and viability under chilled conditions [105]. Furthermore, cross-resistance or “halo effects” may arise, where pathogens that have adapted to one type of stress (e.g., a high temperature) may also exhibit enhanced tolerance to other environmental challenges (e.g., sanitizers, desiccation). For instance, the upregulation of general stress response regulators, like the alternative sigma factor σ^B^ in *Listeria monocytogenes*, can confer protection against multiple stressors, including heat, cold, acid, and oxidative conditions [106]. By understanding the specific molecular and physiological adaptations of foodborne pathogens, food safety professionals can develop more targeted and effective control measures to mitigate the risks posed by these resilient microorganisms across the entire food supply chain.

Certain foods possess natural antimicrobial properties that can hinder the growth of pathogens. For example, plant-based foods, such as fruits and vegetables, are rich in a diverse array of phytochemicals, including phenolic compounds, terpenes, and glucosinolates, which exhibit potent antimicrobial activities [107]. As observed in this review, resilient bacteria such as *Listeria monocytogenes*, *Salmonella*, and *Escherichia coli* can survive for only a few weeks on foods with antimicrobial properties such as yellow onions, kale, black carrot juice, cauliflower, bell peppers, romaine lettuce, iceberg lettuce, perilla leaves, and broccoli. Plant-derived antimicrobials can be integrated into food processing and packaging technologies. This could involve the development of active food packaging materials, the formulation of antimicrobial coatings, or the strategic incorporation of plant extracts and essential oils into food products.

Moreover, the pH of food can affect pathogen survival; however, only a few studies have explored this phenomenon. Foods that possess a low pH (high acidity), such as citrus fruits and vinegar, can impede the growth of most pathogens. However, *Escherichia coli* O157:H7 has the unique ability to tolerate and adapt to acidic environments, enabling it to thrive better in acidic foods and beverages [108]. On the other hand, foods with relatively high pH values (low acidity), including meats and dairy products, may create an environment where most pathogens can survive and multiply. Similarly, the composition of nutrients in food also affects the survival of pathogens. Foods rich in protein, such as meats, boiled-in-bag eggs, chocolate protein drinks, whey protein powder, poultry, food and feed ingredients, and seafood, provide an abundant source of nutrients for pathogens to grow and multiply, thereby increasing their survival period and the risk of contamination. Water activity, which refers to the available water content in food, also plays a role in pathogen survival [109]. Foods with a water activity greater than 0.95 provide a supportive environment for the growth of pathogens [110]. Foods with high water activity include raw meats, fresh produce, fruits, and vegetables. However, foods with low water activity, such as hazelnuts, chia seeds, green beans, corn, peanut butter, whey protein powder, nuts, and dehydrated products, were found to have increased survival rates of *Salmonella*, *Escherichia coli* O157:H7, and *Listeria monocytogenes* compared with foods with high water activity. Low-moisture foods (LMFs) are often associated with outbreaks caused by norovirus and hepatitis A virus. Examples include a norovirus outbreak in 2008 in Korea, where dry radish was identified as the source of 117 symptomatic infections [111]; a norovirus outbreak in 2017 in Japan, involving 2094 cases linked to dry seaweed [112]; and a hepatitis outbreak in Australia, where sun-dried tomatoes were identified as vehicles [113].

Pathogens have the potential to spread from one food to another through cross-contamination. The cross-contamination of ready-to-eat foods, such as salads or fruits, with raw meat can lead to the survival of pathogens, posing a significant risk to human health [96]. Throughout the entire food production process, from primary production to secondary processing, the risk of contamination by persistent foodborne pathogens remains a concern. To prevent this, it is crucial to maintain strict separation between raw and cooked foods, utilize separate cutting boards and utensils, practice good hygiene, and follow good agricultural practices that reduce the contamination and cross-contamination of both crop and animal products. Proper food handling and storage, adherence to the recommended temperatures, good manufacturing practices, and hazard analyses and maintaining cleanliness throughout the food preparation process are all important steps to ensure food safety [114,115]. In addition, specific food processing methods, including cooking, pasteurization, and canning, are effective in eliminating or reducing the presence of pathogens. Cooking food at the appropriate temperatures can effectively kill most pathogens, rendering this food safe for consumption. For example, cooking meat to an internal temperature of 165 °F (74 °C) helps eliminate pathogens such as *Salmonella* and *Escherichia coli* [116,117]. Pasteurization, a heat treatment process commonly used for liquids such as milk and juice, also aids in destroying pathogens while preserving the quality of the product [118]. Importantly, these measures are crucial for minimizing the risk of foodborne illnesses, regardless of the specific type or persistence duration of the pathogens involved. Therefore, regular monitoring, testing, the use of validated process controls, and continuous education and training of food handlers are crucial to ensure the implementation of proper food safety protocols and prevent the persistence of pathogens on food.

While this review provides a comprehensive overview of bacterial and viral pathogen survival and persistence, it also reveals a notable knowledge gap regarding the survival and persistence duration of protozoan parasites and fungi on food contact surfaces, highlighting the need for further research to develop standardized methodologies for assessing the persistence of a broader range of protozoan parasites, such as *Giardia lamblia*, *Toxoplasma* spp., and *Cyclospora* spp., as well as fungi, such as *Aspergillus* spp., *Penicillium* spp., and *Fusarium* spp., on different food contact surface materials. Moreover, the molecular mechanisms and physiological adaptations that allow certain pathogens to persist for extended periods on food-related surfaces remain largely unexplored. Gaining a deeper understanding of these processes could inform the development of innovative strategies for pathogen control, such as the use of antimicrobial coatings or novel disinfection technologies. Finally, the influence of environmental factors, such as temperature, humidity, and the presence of organic matter, on pathogen persistence requires further exploration. While this review highlighted the general trends, a more comprehensive understanding of the complex interactions between pathogens, surface materials, and environmental conditions could inform the development of more effective cleaning and disinfection protocols tailored to specific food processing environments.

### Strengths and Limitations

This review followed the PRISMA guidelines for a thorough search and included data from multiple studies encompassing a wide range of pathogens, including bacteria, viruses, and protozoan parasites. Furthermore, this review considered the influence of environmental factors, particularly temperature, on pathogen survival and persistence. This valuable information can be utilized to shape food safety protocols and guidelines, leading to more effective measures for control and prevention. However, this review has several limitations. There was a greater focus on bacterial isolates, potentially resulting in an underrepresentation of other pathogens, including viruses, fungi, and parasites. Another limitation is the possibility of publication bias, where studies demonstrating longer pathogen persistence may have been more likely to be published, whereas those showing shorter persistence or nonsignificant results may have been overlooked or unpublished. Similarly, reporting bias may exist, as studies selectively report certain aspects of pathogen persistence, possibly omitting negative or inconclusive results. These biases could impact the overall conclusions drawn from the review.

## 5. Conclusions

This review offers important insights into the survival durations of pathogens in food, which have significant implications for public health and the food industry’s ability to ensure the safety of the global food supply. Bacterial pathogens, particularly *Listeria monocytogenes*, *Salmonella*, and *Escherichia coli*, demonstrate a remarkable ability to survive and persist for extended periods, making them a major concern in foodborne illnesses. The limited research on protozoan parasites and the absence of studies on fungi highlight the need for further research to understand their persistence and impact on food safety. Environmental factors such as temperature and humidity play crucial roles in pathogen survival, with some pathogens showing resilience to both high and low temperatures. By bridging the gaps between different scientific disciplines, the power of genomics, environmental science, and advanced analytical techniques can be leveraged to unravel the complex and interconnected mechanisms driving pathogen adaptation, survival, and persistence. Understanding these dynamics is essential for developing effective control strategies to mitigate the risks associated with foodborne pathogens and ensure the safety of food products from farm to fork.

## Figures and Tables

**Table 1:** Average survival/persistence duration of bacteria.

Survival/Persistence	Mean Duration (Days)	Mean Duration (Days) ± SD
Persistence	246.04	21.50, 470.58
Survival	15.69	2.35, 33.73

**Table 2:** Bacterial survival and persistence across various temperature categories.

Temperature Category	Average Duration (Days)
Below Freezing (<0 °C)	96.50
Cold (0–10 °C)	55.25
Moderate (10–25 °C)	67.92
Warm (>25 °C)	316.71
Unknown	35

**Table 3:** Virus survival time period range in different temperature conditions.

Temperature Category	Duration Range (Weeks/Days)	Virus	References
Frozen temperatures (<0 °C)	15 days	Poliovirus	[86]
Low and refrigerated temperatures (0–10 °C)	≤4 weeks and 30 days	Hepatitis A virus Feline calicivirus Norovirus Bacteriophage phi 6 Murine hepatitis virus Transmissible gastroenteritis virus Poliovirus Porcine sapovirus	[77,78,79,80,86]
Room temperature (10–24 °C)	≤6 weeks	Feline calicivirus Human norovirus Hepatitis A virus Bacteriophage MS2 Severe acute respiratory syndrome coronavirus 2 (SARS-CoV-2) Middle East respiratory syndrome (MERS-CoV) Murine norovirus	[79,81]
Warm (≥25 °C)	2 weeks	Hepatitis A virus Murine norovirus	[76]

## Appendix A

**Table A1:** Bacterial survival and persistence in various food types.

Bacteria	Sample	Duration	Condition	Survival/Persistence	Reference
*Campylobacter jejuni*	Unpasteurized milk	21 days	4 °C	Survival	Doyle et al. [20]
*Campylobacter jejuni* ST-883	Raw milk	4–6 days	-	Survival	Jaakkonen et al. [21]
*Cronobacter*	Powdered infant formula (PIF)	3 months	-	Persistence	Bennour Hennekinne et al. [22]
*Cronobacter muytjensii*	Infant wheat-based formulas reconstituted with water, milk, grape juice, or apple juice	24 h	4, 25, or 37 °C	Survival	Osaili et al. [23]
*Cronobacter sakazakii*	Infant wheat-based formulas reconstituted with water, milk, grape juice, or apple juice	24 h	4, 25, or 37 °C	Survival	Osaili et al. [23]
*Vibrio cholerae* (*El Tor*)	Parsley	24 h	-	Survival	Sechter et al. [24]
*Vibrio cholerae* (*El Tor*)	Tomatoes and carrots	24 to 30 h	-	Survival	Sechter et al. [24]
*Vibrio cholerae* (*El Tor*)	Cucumbers, peppers, and okra	24–48 h	-	Survival	Sechter et al. [24]
*Vibrio cholerae* (*El Tor*)	Lettuce	2–3 days	-	Survival	Sechter et al. [24]
*Enterococcus faecalis*	Poultry and cattle feed	7 days	22 °C, 65% relative humidity	Survival	Channaiah et al. [25]
*Escherichia coli*	Raw carrot and cucumber juice	10 days	20 °C	Survival	Van Beeck et al. [26]
*Escherichia coli* O157:H7	Bruised and unbruised tomatoes	7 days	10 and 20 °C	Survival	Tokarskyy et al. [27]
*Escherichia coli* O157:H7	In-shell hazelnuts	12 months	24 ± 1 °C, 40 ± 3% relative humidity	Persistence	Feng et al. [28]
*Escherichia coli* O157:H7	Black carrot juice	7 days	4 and 37 °C	Survival	Degirmenci et al. [29]
*Escherichia coli* O157:H7	The rhizosphere and leaf surfaces of lettuce	7–21 days	-	Survival	Mark Ibekwe et al. [30]
*Escherichia coli* O157:H7	Lettuce cultivars	12 days	-	Survival	Erickson et al. [32]
*Escherichia coli* O157:H7	Cabbage cultivars	9 days	-	Survival	Erickson et al. [31]
*Escherichia coli* O157:H7	Vegetables (romaine lettuce, iceberg lettuce, perilla leaves, and sprouts)	7 days	15 °C	Survival	Tian et al. [33]
*Escherichia coli* O157:H7	Grape pulp	30 days	4 °C, pH 2.51–3.26	Survival	Marques et al. [34]
*Escherichia coli* O157:H7	Whole and diced yellow onions (Allium cepa)	6 days	4 °C, 30–50% relative humidity	Survival	Lieberman et al. [35]
*Escherichia coli* O157:H7	Walnut kernels	3 weeks to more than 1 year	23 °C	Persistence	Blessington et al. [36]
*Escherichia coli* O157:H7	Raw peanut and pecan kernels	365 days	−24 ± 1, 4 ± 2, and 22 ± 1 °C	Persistence	Brar et al. [37]
*Escherichia coli* O157:H7	Cheddar cheese whey	21 days	4, 10 or 15 °C, pH 5.5	Survival	Marek et al. [38]
*Escherichia coli* O157:H7	Lamb meat	12 days	4 and 12 ± 1 °C	Survival	Barrera et al. [39]
*Escherichia coli* O157:H7	Galotyri cheese	28 days	4 and 12 °C	Survival	Lekkas et al. [40]
*Escherichia coli* O157:H7	Raw goat milk lactic cheeses	42 days	-	Survival	Vernozy-Rozand et al. [41]
*Escherichia coli* O157:H7	Cheese	90 days	-	Persistence	Maher et al. [42]
*Helicobacter suis*	Retail pork samples	at least 48 h	-	Survival	De Cooman et al. [43]
*Listeria monocytogenes*	Whole mango	28 days	12 ± 2 °C	Survival	Saha et al. [44]
*Listeria monocytogenes*	Mixed vegetables (containing green beans, corn, and peas)	12 months	−18 or −10 °C	Persistence	Fay et al. [45]
*Listeria monocytogenes*	Edible seaweed	7 days	4, 10, and 22 °C	Survival	Akomea-Frempong et al. [46]
*Listeria monocytogenes*	Raw carrot and cucumber juice	8 days	20 °C	Survival	Van Beeck et al. [26]
*Listeria monocytogenes*	Black carrot juice	7 days	4 and 37 °C	Survival	Degirmenci et al. [29]
*Listeria monocytogenes*	Vegetables (romaine lettuce, iceberg lettuce, perilla leaves, and sprouts)	7 days	15 °C	Survival	Tian et al. [33]
*Listeria monocytogenes*	Walnut kernels	3 weeks to more than 1 year	23 °C	Persistence	Blessington et al. [36]
*Listeria monocytogenes*	Raw peanut and pecan kernels	28 or 365 days	−24 ± 1, 4 ± 2, and 22 ± 1 °C	Persistence	Brar et al. [37]
*Listeria monocytogenes*	Chickpeas, sesame seeds, pine nuts, and black pepper kernels	180 days	25 °C, 25, 45, and 75% relative humidity	Persistence	Salazar et al. [47]
*Listeria monocytogenes*	Nut-, seed-, legume-, and chocolate-containing butters	6 months	5 or 25 °C	Persistence	Fay et al. [48]
*Listeria monocytogenes*	Chocolate liquor, corn flakes, and shelled, dry-roasted pistachios	336 days	4 and 25 °C, 35%relative humidity	Persistence	Ly et al. [49]
*Listeria monocytogenes*	Dried apples, raisins, and dried strawberries	336 days	4 and 23 °C	Persistence	Cuzzi et al. [50]
*Listeria monocytogenes*	Fresh strawberries	4 weeks	−20 ± 2 °C	Survival	Flessa et al. [51]
*Listeria monocytogenes*	Bell peppers	14 days	4 °C	Survival	Moreira et al. [52]
*Listeria monocytogenes*	Cantaloupe rind	7 days	24 °C	Survival	Moreira et al. [52]
*Listeria monocytogenes*	Kale, cauliflower, and broccoli	6 days	13 °C	Survival	Moreira et al. [52]
*Mycobacterium avium subsp. paratuberculosis* (*Map*)	Ultrafiltered white cheese	60 days	-	Survival	Hanifian [53]
*Mycobacterium paratuberculosis*	Cheddar cheese	27 weeks	-	Persistence	Donaghy et al. [54]
*Salmonella*	Whole mango	28 days	12 ± 2 °C	Survival	Saha et al. [44]
*Salmonella*	Bruised and unbruised tomatoes	7 days	10 and 20 °C	Survival	Tokarskyy et al. [27]
*Salmonella*	Peanut oil	96 ± 8 days	-	Persistence	Fong et al. [55]
*Salmonella*	Chia seeds	94 ± 46 days	-	Persistence	Fong et al. [55]
*Salmonella*	Peanut shell	42 ± 49 h	-	Survival	Fong et al. [55]
*Salmonella*	Edible seaweeds	7 days	4, 10, and 22 °C	Survival	Akomea-Frempong et al. [46]
*Salmonella*	Lettuce cultivars	12 days	-	Survival	Erickson et al. [32]
*Salmonella*	Cabbage cultivars	9 days	-	Survival	Erickson et al. [31]
*Salmonella*	Dry- and wet-inoculated sucrose	52 weeks	5 and 25 °C	Persistence	Beuchat et al. [56]
*Salmonella*	Boil-in-bag eggs	36 months	40 °C	Persistence	Flock et al. [57]
*Salmonella*	Chocolate protein drink and peanut butter	12 months	40 °C	Persistence	Flock et al. [57]
*Salmonella*	Whole and diced yellow onions (Allium cepa)	6 days	4 °C, 30–50% relative humidity	Survival	Lieberman et al. [35]
*Salmonella*	Walnut kernels	3 weeks to more than 1 year	23 °C	Persistence	Blessington et al. [36]
*Salmonella*	Raw peanut and pecan kernels	28 or 365 days	−24 ± 1, 4 ± 2, and 22 ± 1 °C	Persistence	Brar et al. [37]
*Salmonella*	Dehydrated garlic flakes	88 days	25 °C, ambient relative humidity	Survival	Zhang et al. [58]
*Salmonella*	Ground black pepper (Piper nigrum)	45 or 100 days	25 or 35 °C, high relative humidity	Persistence	Keller et al. [59]
*Salmonella*	Ground ginger	170 or 365 days	25 and 37 °C, 33% (low) and 97% (high) relative humidity	Persistence	Gradl et al. [60]
*Salmonella*	Whole almond kernels	28 days	35, 22, 4, or −18 °C	Survival	Xu et al. [61]
*Salmonella*	Strawberries, cranberries, date paste, and raisins	182–242 days	4 and 25 °C	Persistence	Beuchat et al. [62]
*Salmonella*	Ground black pepper (Piper nigrum)	8 months	25 and 35 °C, ambient humidity	Persistence	Keller et al. [59]
*Salmonella dublin*	Beef–pork pepperoni	42–43 days	-	Survival	Smith et al. [63]
*Salmonella enterica*	Tomato	14 days	−16.7 °C	Survival	Zhou et al. [64]
*Salmonella enterica*	Raw carrot and cucumber juice	10 days	20 °C	Survival	Van Beeck et al. [26]
*Salmonella enterica*	Whey protein powder	6 months	36 °C	Persistence	Farakos et al. [65]
*Salmonella enterica*	Catfish mucus	63 days	10–22 °C	Survival	Dhowlaghar et al. [66]
*Salmonella enterica serovar enteritidis* ATCC 13076	Colonial cheese	28 days	-	Survival	Degenhardt et al. [67]
*Salmonella enterica serovar typhimurium*	Vegetables (romaine lettuce, iceberg lettuce, perilla leaves, and sprouts)	7 days	15 °C	Survival	Tian et al. [33]
*Salmonella enteritidis* PT 30	Almond kernels	68 weeks	23 ± 0.5 °C	Persistence	Limcharoenchat et al. [68]
*Salmonella enteritidis* PT 30	Whole Nonpareil variety almonds	48 weeks	4 or 23 °C	Persistence	Abd et al. [69]
*Salmonella tennessee*	Peanut butter	14 days	-	Survival	Matak et al. [70]
*Salmonella typhimurium*	Peanut butter	14 days	-	Survival	Matak et al. [70]
*Salmonella typhimurium*	Black carrot juice	7 days	4 and 37 °C	Survival	Degirmenci et al. [29]
*Salmonella typhimurium*	Pacific oyster	30 days	-	Survival	Chakroun et al. [71]
*Salmonella typhimurium*	Cheddar, Swiss, and mozzarella cheeses	2 months	5 °C, pH 5.8	Survival	Leyer et al. [72]
*Shiga toxin-producing Escherichia coli* (*STEC*)	Fermented sausages	28 days	-	Survival	Böhnlein et al. [73]
*Shiga toxin-producing Escherichia coli* O157:H7. STEC O157	Korean-style kimchi	8 weeks	4 °C	Survival	Gill et al. [74]
*Shigatoxigenic Escherichia coli* (*STEC*)	Edible seaweeds	7 days	4, 10, and 22 °C	Survival	Akomea-Frempong et al. [46]
*Staphylococcus aureus*	Vegetables (romaine lettuce, iceberg lettuce, perilla leaves, and sprouts)	7 days	15 °C	Survival	Tian et al. [33]
*Vibrio*	Edible seaweed	7 days	4, 10, and 22 °C	Survival	Akomea-Frempong et al. [46]
*Vibrio cholerae* (*El Tor*)	Parsley	24 h	-	Survival	Sechter et al. [24]
*Vibrio cholerae* (*El Tor*)	Tomatoes and carrots	24 to 30 h	-	Survival	Sechter et al. [24]
*Vibrio cholerae* (*El Tor*)	Cucumbers, peppers, and okra	24–48 h	-	Survival	Sechter et al. [24]
*Vibrio cholerae* (*El Tor*)	Lettuce	2–3 days	-	Survival	Sechter et al. [24]
*Vibrio vulnificus*	Oysters	2 weeks	Refrigeration conditions	Survival	Wood et al. [75]

**Table A2:** Survival and persistence of viruses on various food types.

Viruses	Sample	Duration	Condition	Survival/Persistence	Reference
*Bacteriophage MS2*	Oysters, fresh peppers	2 weeks	4, 15, 25, and 40 °C, 50% and 70% relative humidity	Survival	Lee et al. [76]
*Bacteriophage MS2*	Eastern oysters (Crassostrea virginica)	6 weeks	7, 15, or 24 °C	Survival	Kingsley et al. [84]
*Bacteriophage phi 6*	Meat, fish products	30 days	refrigerated and frozen temperatures	Survival	Bailey et al. [77]
*Calicivirus* (*CV*)	Lettuce leaves	7–14 days	4 °C	Survival	Esseili et al. [78]
*Feline calicivirus* (*FCV*)	Cereal, chocolate, pistachios	4 weeks	room temperature	Survival	Nasheri et al. [79]
*Feline calicivirus* (*FCV*)	Marinated mussels	4 weeks	4 °C	Survival	Hewitt et al. [80]
*Hepatitis A virus* (*HAV*)	Alfalfa seeds	50 days	22 °C	Survival	Wang et al. [81]
*Hepatitis A virus* (*HAV*)	Cereal, chocolate, pistachios	4 weeks	room temperature	Survival	Nasheri et al. [79]
*Hepatitis A virus* (*HAV*)	Marinated mussels	4 weeks	4 °C, pH 3.75	Survival	Hewitt et al. [80]
*Hepatitis A virus* (*HAV*)	Oysters or the surface of fresh peppers	2 weeks	4, 15, 25, and 40 °C, 50% and 70% relative humidity (RH)	Survival	Lee et al. [76]
*Hepatitis A virus* (*HAV*)	Lettuce	9 days	4 °C	Survival	Croci et al. [82]
*Hepatitis A virus* (*HAV*)	Carrot	4 days	4 °C	Survival	Croci et al. [82]
*Hepatitis A virus* (*HAV*)	Fennel	7 days	4 °C	Survival	Croci et al. [82]
*Human norovirus* (*NoV*)	Cereal, chocolate, pistachios	4 weeks	room temperature	Survival	Nasheri et al. [79]
*Middle East respiratory syndrome* (*MERS-CoV*)	Apples, tomatoes, cucumbers	72 hrs	22 °C 30–40% relative humidity	Survival	Blondin-Brosseau et al. [83]
*Murine hepatitis virus* (*MHV*)	Meat and fish products	30 days	refrigerated and frozen temperatures	Survival	Bailey et al. [77]
*Murine norovirus* (*MNV*)	Alfalfa seeds	50 days	22 °C	Survival	Wang et al. [81]
*Murine norovirus* (*MNV*)	Cereal, chocolate, pistachios	4 weeks	room temperature	Survival	Nasheri et al. [79]
*Murine norovirus* (*MNV*)	Lettuce leaves	7–14 days	4 °C	Survival	Esseili et al. [78]
*Murine norovirus* (*MNV*)	Oysters or the surface of fresh peppers	2 weeks	4, 15, 25, and 40 °C, 50% and 70% relative humidity	Survival	Lee et al. [76]
*Noroviruses* (*NoV*)	Lettuce and turkey	at least 10 days	7 °C	Survival	Lamhoujeb et al. [85]
*Noroviruses* (*NoV*)	Marinated mussels	4 weeks	4 °C	Survival	Hewitt et al. [80]
*Poliovirus*	Lettuce, green onion, white cabbage	15 days	4 °C	Survival	Kurdziel et al. [86]
*Poliovirus*	Strawberries	15 days	−20 °C	Survival	Kurdziel et al. [86]
*Poliovirus*	Raspberries	9 days	4 °C	Survival	Kurdziel et al. [86]
*Porcine Delta Corona Virus* (*PDCoV*)	Feed ingredient matrices	56 days	-	Survival	Trudeau et al. [87]
*Porcine Epidemic Diarrhea Virus* (*PEDV*)	Feed ingredient matrices	56 days	-	Survival	Trudeau et al. [87]
*Porcine sapovirus* (*SaV*)	Lettuce leaves	7–14 days	4 °C	Survival	Esseili et al. [78]
*Severe acute respiratory syndrome coronavirus 2* (*SARS-CoV-2*)	Ready-to-eat deli items, fresh produce, and meats (including seafood)	21 days	-	Survival	Jia et al. [88]
*Severe acute respiratory syndrome coronavirus 2* (*SARS-CoV-2*)	Apples, tomatoes, cucumbers	72 hrs	22 °C, 30–40% relative humidity	Survival	Blondin-Brosseau et al. [83]
*Severe acute respiratory syndrome coronavirus 2* (*SARS-CoV-2*)	Stew-cut beef and ground beef	2 days	-	Survival	Featherstone et al. [89]
*Transmissible gastroenteritis virus* (*TGEV*)	Feed ingredient matrices	56 days	-	Survival	Trudeau et al. [87]
*Transmissible gastroenteritis virus* (*TGEV*)	Meat and fish products	30 days	refrigerated and frozen temperatures	Survival	Bailey et al. [77]
